# Non-traditional Lipid Parameters as Potential Predictors of Asymptomatic Intracranial Arterial Stenosis

**DOI:** 10.3389/fneur.2021.679415

**Published:** 2021-08-31

**Authors:** Jiahuan Guo, Anxin Wang, Yu Wang, Xinmin Liu, Xiaoli Zhang, Shouling Wu, Xingquan Zhao

**Affiliations:** ^1^Department of Neurology, Beijing Tiantan Hospital, Capital Medical University, Beijing, China; ^2^China National Clinical Research Center for Neurological Diseases, Beijing Tiantan Hospital, Capital Medical University, Beijing, China; ^3^Research Unit of Artificial Intelligence in Cerebrovascular Disease, Chinese Academy of Medical Sciences, Beijing, China; ^4^Department of Cardiology, Kailuan Hospital, North China University of Science and Technology, Tangshan, China; ^5^Center of Stroke, Beijing Institute for Brain Disorders, Beijing, China

**Keywords:** asymptomatic intracranial atherosclerosis, lipid parameters, predictor, epidemiology, prevalence

## Abstract

**Background:** Intracranial arterial stenosis (ICAS) is a common cause of stroke. Identifying effective predictors of ICAS that could be easily obtained in clinical practice is important. The predictive values of serum individual lipid parameters have been well-established. In recent years, several non-traditional lipid parameters demonstrated greater predictive values for cardiovascular disease and ischemic stroke than traditional individual lipid parameters. However, their effects on asymptomatic ICAS (aICAS) are less clear. Therefore, we sought to observe the effects of non-traditional lipid parameters on aICAS.

**Methods:** We enrolled 5,314 participants from the Asymptomatic Polyvascular Abnormalities in Community study. Asymptomatic ICAS was detected by transcranial Doppler ultrasonography (TCD). Non-traditional lipid parameters, including non-high-density lipoprotein cholesterol (non-HDL-C), the triglycerides/high-density lipoprotein cholesterol ratio (TG/HDL-C), atherogenic coefficient (AC), atherogenic index of plasma, and Castelli's risk index (CRI) were measured. We used multivariable logistic analysis to assess the association of different lipid parameters with aICAS; a trend test and subgroup analyses were also performed.

**Results:** In total, 695 of 5,314 participants had aICAS in this study. For the comparison of the highest to the lowest tertile, the multivariable-adjusted odds ratios (ORs) (95% CIs) were 1.78 (1.39–2.27) (*p* trend < 0.001) for non-HDL-C, 1.48 (1.18–1.85) (*p* trend = 0.004) for the AC, 1.48 (1.18–1.85) (*p* trend = 0.004) for CRI-I, and 1.34 (1.09–1.66) (*p* trend = 0.032) for CRI-II. Subgroup analyses showed significant interactions between the AC, CRI-I, and diabetes.

**Conclusions:** This large community-based study showed that non-HDL-C, AC, CRI-I, and CRI-II were significantly associated with increased prevalence of aICAS.

## Introduction

Intracranial arterial stenosis (ICAS) is one of the most common causes of stroke, especially in Asian, Hispanic, and African populations (1). In fact, ICAS is estimated to account for 30–50% of strokes and more than 50% of transient ischemic attacks in Asian people, and the burden of stroke due to ICAS is likely to continuously grow (2, 3). In addition, ICAS also contributes to the recurrence of stroke (4). In this regard, identifying and managing risk factors for asymptomatic ICAS (aICAS) in an early stage are crucial steps for slowing the progression and lowering the risk of further vascular events.

Low-density lipoprotein cholesterol (LDL-C) is well-known as a key factor in cerebral vascular disease. Large-scale randomized clinical trials have revealed that lowering cholesterol concentrations with statins reduces the risk of ischemic stroke (5). Other lipid and lipoprotein biomarkers, such as total cholesterol (TC), high-density lipoprotein cholesterol (HDL-C), and triglycerides (TG), have also been identified as risk factors for ischemic stroke over the past decades (6, 7). Thus, it is reasonably hypothesized that lipid parameters could be predictors of aICAS. A better understanding of the relationship between lipid parameters and aICAS may facilitate a clearer perception of the underlying pathophysiology of aICAS and help identify the most effective biomarker to target with lipid-modifying therapeutics target as well.

In recent years, evidence has increasingly shown that traditional individual lipid parameters may not be the best predictors of cardiovascular and cerebrovascular disease risk conferred by atherogenic lipoproteins. Several non-traditional lipid parameters, including non-HDL-C, the TG/HDL-C ratio, atherogenic index of plasma [AIP; calculated as log_10_(TG/HDL-C)], atherogenic coefficient (AC; calculated as non-HDL-C/HDL-C), Castelli's risk index-I (CRI-I; calculated as TC/HDL-C), and Castelli's risk index-II (CRI-II; calculated as LDL-C/HDL-C) have greater predictive values, as they could better reflect the overall interaction between lipid/lipoprotein fractions (8–10).

However, studies for the association between traditional lipid parameters and aICAS have shown variable results (11–13). Additionally, few studies have evaluated the relationship between non-traditional lipid parameters and aICAS. Therefore, we aim to relate a variety of plasma measurements, including traditional lipid parameters (TC, TG, LDL-C, and HDL-C) and non-traditional measurements (non-HDL-C, TG/HDL-C, AIP, AC, and CRI-I, CRI-II), to the prevalence of aICAS.

## Materials and Methods

### Study Design and Population

The Asymptomatic Polyvascular Abnormalities in Community (APAC) study is a subset of the Kailuan study, which is a community-based, prospective, observational study that aims to investigate the epidemiology of asymptomatic polyvascular abnormalities in Chinese adults. The details of the Kailuan study design have been published previously (14). Briefly, a total of 5,440 participants met the inclusion criteria of the APAC study as follows: (1) aged 40 years or over; (2) no history of coronary heart disease, stroke, or transient ischemic attack at baseline; and (3) absence of neurologic deficits related to stroke. Our study was a cross-sectional analysis assessing the association between different lipid parameters and the prevalence of aICAS. After excluding 53 participants with incomplete TG, TC, HDL-C, or transcranial Doppler ultrasonography (TCD) data at baseline and 73 participants who were taking lipid-lowering agents, we finally included 5,314 participants in total (Supplementary Figure 1).

### Measurement of Calculation of Lipid Parameters

Blood samples were collected under fasting conditions and analyzed within 4 h of preparation using an autoanalyzer (Hitachi 747; Hitachi, Tokyo, Japan) at the central laboratory of Kailuan Hospital. The concentration of fasting blood glucose (FBG) and blood lipid measurements in this study have been described in detail in a previous study (15). Non-traditional lipid parameters were calculated as follows: non-HDL-C was defined as subtracting serum HDL-C concentration from TC. Atherogenic coefficient was calculated as non-HDL-C/HDL-C; AIP was calculated as log_10_(TG/HDL-C); CRI-I was calculated as TC/HDL-C; and CRI-II was calculated as LDL-C/HDL-C (10, 16, 17).

### Measurement of Potential Covariates at Baseline

Data on demographic variables, education level, lifestyle habits, and medical history were collected with a questionnaire by trained investigators. A subject was considered a current smoker if he or she smoked at least one cigarette per day. Current alcohol use refers to alcohol intake of at least 90 g of liquor a day for more than a year for men and 45 g for women. Active physical activity was defined as four or more times per week and 20 or more minutes at a time. Body mass index (BMI) was calculated as follows: BMI = weight (kg)/square of height (m^2^). Diabetes mellitus was defined as a FBG level ≥7.0 mmol/L (126 mg/dl), a previous history of diabetes, or current use of antidiabetic agents. Blood pressure was measured on the left arm in seated position using a mercury sphygmomanometer following the standard recommended procedures. Two measures of each systolic blood pressure and diastolic blood pressure were taken after participants had rested for at least 5 min. The average of two readings was used, if the results of the two readings differed by more than 5 mmHg, an additional reading was taken and the average of the three readings was used. Hypertension was defined as a systolic blood pressure ≥140 mmHg or a diastolic blood pressure ≥90 mmHg, a previous history of hypertension, or current use of antihypertensive agents. Dyslipidemia was defined as a TC level ≥5.17 mmol/L (220 mg/dl), a TG level ≥1.7 mmol/L (150 mg/dl), a previous history of dyslipidemia, or current use of lipid-lowering agents.

### Measurement of ICAS

Transcranial Doppler ultrasonography is a non-invasive and reliable tool for diagnosing ICAS and shows satisfactory agreement with magnetic resonance angiography (MRA) and computed tomography angiography (CTA). In our study, two experienced technicians, blinded to the information of the participants, performed TCD with portable devices (EME Companion, Nicolet, Madison, WI, USA) to detect ICAS. According to published criteria, ICAS was defined by the peak systolic flow velocity as follows: >140 cm/s for the middle cerebral artery, >120 cm/s for the anterior cerebral artery, >100 cm/s for the posterior cerebral artery and the vertebral-basilar artery, or >120 cm/s for the siphon internal carotid artery. The age of the participant, presence of turbulence or a musical sound, and whether the abnormal velocity was segmental were also taken into consideration. ICAS was diagnosed if stenosis or occlusion was found in one of the intracranial arteries mentioned above (18).

### Statistical Analysis

We classified the participants into three groups according to the tertiles of each lipid parameter, with the lowest tertile being the reference group. Continuous variables are described by medians and interquartile range (IQR) because of skewed distributions. Categorical variables are presented as frequencies and percentages. The Wilcoxon test was used for continuous variables to compare group differences, and the chi-square test was used to test categorical variables.

Both univariate and multivariable logistic regression analyses were performed to assess the prevalence of aICAS according to tertiles of lipid parameters by calculating the odds ratios (ORs) and the corresponding 95% confidence intervals (95% CIs). Model 1 was unadjusted. Model 2 was adjusted for age and sex. Model 3 was further adjusted for BMI, education, income, physical activity, smoking status, drinking status, history of hypertension, diabetes, dyslipidemia, antihypertensive agents, and antidiabetic agents. A trend test was used to assess whether there was a dose-dependent relationship between different lipid parameters and aICAS. In addition, subgroup analyses stratified by sex (male and female), age (< 60 and ≥60 years), BMI (< 25 and ≥25 kg/m^2^), diabetes (no and yes), and hypertension (no and yes) were also performed to assess whether there was any significant interaction between these variables and the relationship between the non-traditional lipid parameters and aICAS.

All statistical analyses were conducted using SAS version 9.4 (SAS Institute Inc., Cary, NC, USA). A two-sided *p* < 0.05 was considered statistically significant.

## Results

### Baseline Characteristics

Among the 5,440 participants enrolled in the APAC study, 5,314 participants with complete information were ultimately enrolled in our cross-sectional analysis. The median age was 52.39 years (IQR, 45.62–61.55). Among them, 60.1% of participants (3,193/5,314) were men.

### Correlation Between Different Lipid Parameters and the Prevalence of aICAS

Of the 5,314 participants enrolled in the cross-sectional analysis, 13.1% (695/5,314) were diagnosed with aICAS based on the TCD data at baseline. People who were diagnosed with aICAS at baseline were more likely to be men; be older; have higher systolic blood pressure; have higher levels of FBG, TC, LDL-C, non-HDL-C, AC, CRI-I, and CRI-II; have a lower educational background level; and were more likely to suffer from diabetes, hypertension, and dyslipidemia than those not diagnosed with aICAS at baseline. This group also had higher proportions of current antihypertensive and antidiabetic agent use (Table 1).

**Table 1:** Clinical characteristics between groups with/without aICAS.

**Variable**	**Without aICAS**	**With aICAS**	***P*-value**
*N* (%)	4,619 (86.9)	695 (13.1)	
Age, years	51.72 (45.36–59.91)	59.00 (49.91–72.48)	<0.001
Male, *n* (%)	2,750 (59.5)	443 (63.7)	0.035
High school or above, *n* (%)	2,046 (44.3)	262 (31.7)	0.001
Income >1,000 RMB/m, *n* (%)	3,617 (78.3)	561 (80.7)	0.148
BMI, kg/m^2^	24.72 (22.65–26.99)	24.77 (22.86–27.34)	0.355
Systolic blood pressure, mm Hg	130.0 (118.7–140.0)	140.0 (130.0–156.7)	<0.001
Diastolic blood pressure, mm Hg	80.67 (76.67–90.00)	80.67 (76.67–90.00)	0.061
Current smoker, *n* (%)	1,481 (32.1)	220 (32.0)	0.830
Current alcohol use, *n* (%)	672 (14.6)	82 (11.8)	0.053
Active physical activity, *n* (%)	2,753 (59.6)	2,753 (59.6)	0.226
Diabetes, *n* (%)	484 (10.5)	149 (21.4)	<0.001
Hypertension, *n* (%)	2,065 (44.7)	475 (60.4)	<0.001
Dyslipidemia, *n* (%)	2,176 (47.1)	372 (53.5)	0.002
Antihypertensive agents, *n* (%)	752 (16.3)	256 (36.8)	<0.001
Antidiabetic agents, *n* (%)	227 (4.9)	85 (12.2)	<0.001
FBG, mmol/L	5.20 (4.81–5.72)	5.40 (4.89–6.22)	<0.001
TC, mmol/L	4.91 (4.33–5.59)	5.17 (4.51–5.81)	<0.001
TG, mmol/L	1.29 (0.92–1.92)	1.32 (0.98–1.92)	0.167
HDL-C, mmol/L	1.57 (1.31–1.90)	1.32 (0.98–1.85)	0.051
LDL-C, mmol/L	2.59 (2.16–3.04)	2.70 (2.21–3.15)	0.002
Non-HDL-C, mmol/L	3.29 (2.74–3.97)	3.63 (2.96–4.25)	<0.001
TG/HDL-C	0.83 (0.54–1.31)	0.88 (0.58–1.34)	0.054
AC	2.09 (1.60–2.78)	2.37 (1.76–3.04)	<0.001
AIP	−0.19 (−0.61–0.27)	−0.13 (−0.54–0.29)	0.054
CRI-I	3.09 (2.60–3.97)	3.37 (2.76–4.04)	<0.001
CRI-II	1.63 (1.28–2.04)	1.73 (1.35–2.19)	<0.001

**Figure 1:**
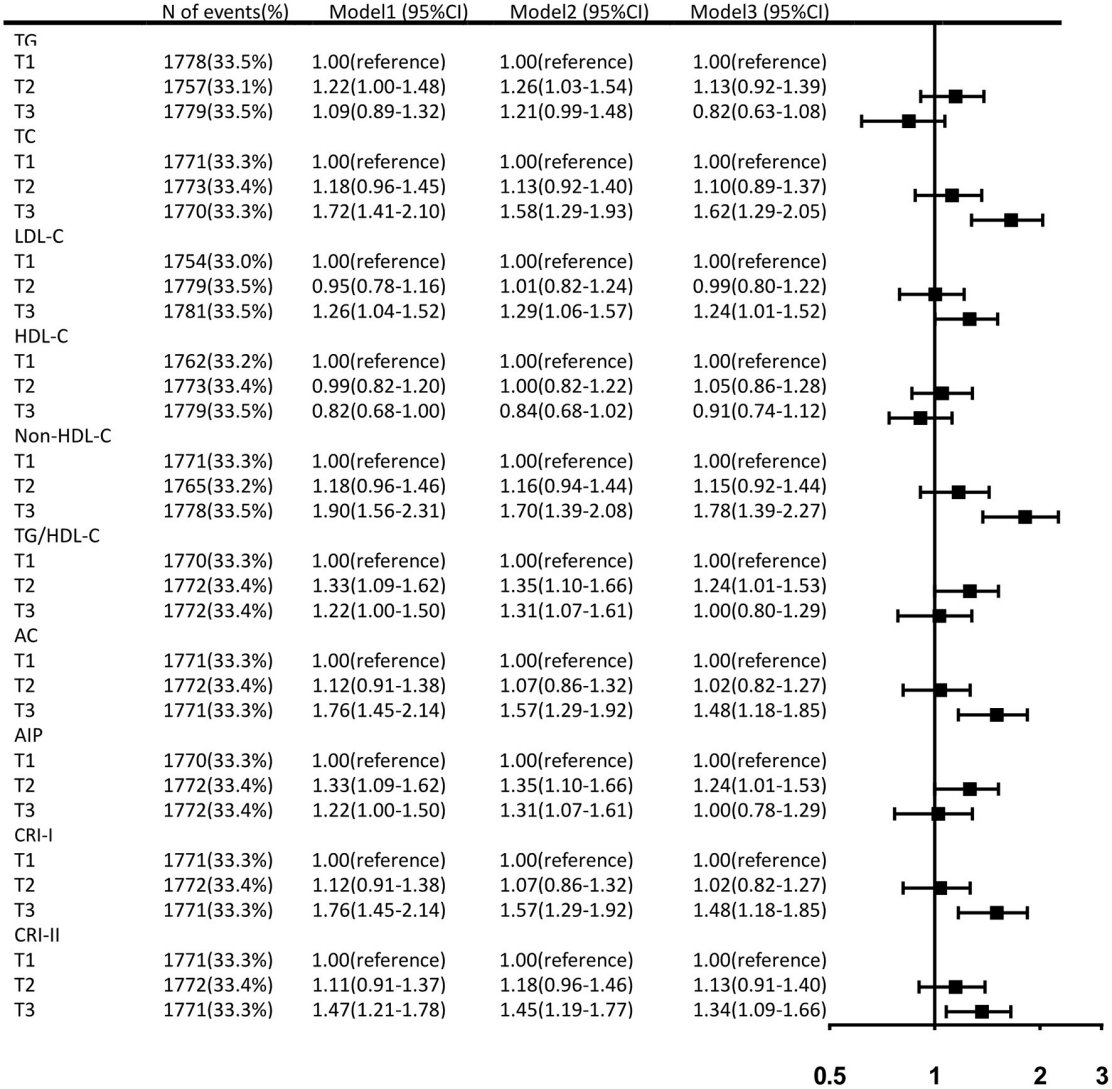
Fully adjusted ORs and 95% CIs of lipid parameters for the prevalence of aICAS. Data were adjusted for age, gender, BMI, education, income, physical activity, smoking status, drinking status, history of hypertension, diabetes, dyslipidemia, antihypertensive agents, and antidiabetic agents.

**Figure 2:**
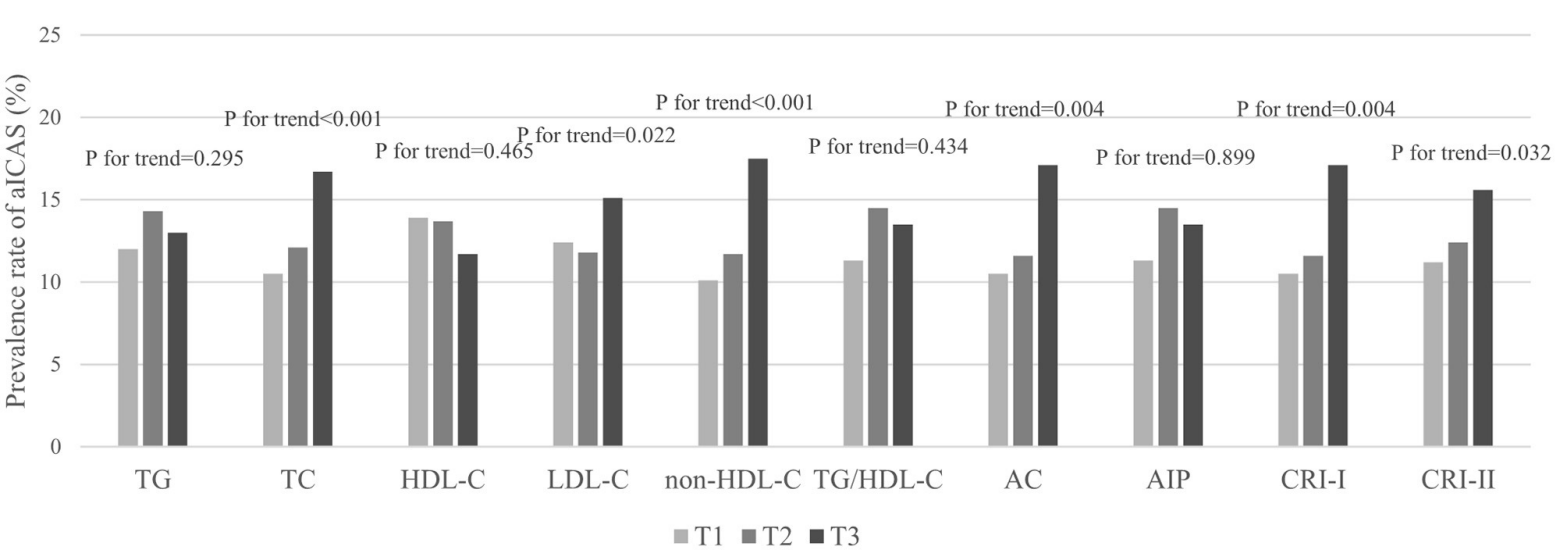
Prevalence rate of aICAS in tertiles of different lipid parameters.

Prior to adjusting for any potential covariates, among the traditional lipid parameters, the highest tertiles of TC (OR, 1.72; 95% CI, 1.41–2.10) and LDL-C (OR, 1.26; 95% CI, 1.04–1.52) were associated with the prevalence of aICAS compared to the lowest tertile. All of the non-traditional parameters we targeted, including non-HDL-C (OR, 1.90; 95% CI, 1.56–2.31), TG/HDL-C (OR, 1.22; 95% CI, 1.00–1.50), AC (OR, 1.76; 95% CI, 1.45–2.14), AIP (OR, 1.22; 95% CI, 1.00–1.50), CRI-I (OR, 1.76; 95% CI, 1.45–2.14), and CRI-II (OR, 1.47; 95% CI, 1.21–1.78) were associated with the prevalence of aICAS. After fully adjusting for potential confounders, the statistical significance of TG/HDL-C and AIP disappeared. A significant association persisted for the highest tertiles of TC (OR, 1.62; 95% CI, 1.29–2.05), LDL-C (OR, 1.24; 95% CI, 1.01–1.52), non-HDL-C (OR, 1.78; 95% CI, 1.39–2.27), AC (OR, 1.48; 95% CI, 1.18–1.85), CRI-I (OR, 1.48; 95% CI, 1.18–1.85), CRI-II (OR, 1.34; 95% CI, 1.09–1.66), and the prevalence of aICAS. Among the traditional and non-traditional lipid parameters, non-HDL-C had the highest OR (Figure 1). The trend test showed that in the fully adjusted model, the prevalence of aICAS was increased with higher tertiles of TC (*p* for trend < 0.001), LDL-C (*p* for trend = 0.022), non-HDL-C (*p* for trend < 0.001), AC (*p* for trend = 0.004), CRI-I (*p* for trend = 0.004), and CRI-II (*p* for trend = 0.032) (Figure 2).

Further subgroup analyses showed a significant interaction between AC and diabetes, and the effect of increased AC on the prevalence of aICAS was stronger in participants with diabetes than in those without diabetes in the fully adjusted model (*p* for interaction = 0.048). The same result was also found between CRI-I and diabetes (*p* for interaction = 0.048) (Table 2). There were no significant interactions in other subgroups including age, sex, BMI, and hypertension (Supplementary Table 1).

**Table 2:** Adjusted ORs with 95% CI of aICAS according to non-traditional lipid parameters, stratified by diabetes.

	**Without diabetes**	**Diabetes**
**TG/HDL-C**		
T1	1	1
T2	1.21(0.96–1.51)	1.53(0.88–2.67)
T3	0.94(0.71–1.24)	1.38(0.76–2.51)
*p* for trend	0.186	0.560
Continuous scale	0.98(0.86–1.13)	1.16(0.86–1.56)
*p*-interaction	0.661	
**AC**		
T1	1	1
T2	0.97(0.76–1.23)	1.49(0.82–2.72)
T3	1.28(1.00–1.65)	3.06(1.66–5.65)
*p* for trend	0.135	0.002
Continuous scale	1.14(1.00–1.29)	1.80(1.33–2.43)
*p*-interaction	**0.048**	
**AIP**		
T1	1	1
T2	1.21(0.96–1.51)	1.53(0.88–2.67)
T3	0.94(0.71–1.24)	1.38(0.76–2.51)
*p* for trend	0.520	0.338
Continuous scale	0.98(0.86–1.13)	1.16(0.86–1.56)
*p*-interaction	0.661	
**CRI-I**		
T1	1	1
T2	0.97(0.76–1.23)	1.49(0.82–2.72)
T3	1.28(1.00–1.65)	3.06(1.66–5.65)
*p* for trend	0.135	0.002
Continuous scale	1.14(1.00–1.29)	1.80(1.33–2.43)
*p*-interaction	**0.048**	
**CRI-II**		
T1	1	1
T2	1.10(0.86–1.39)	1.30(0.78–2.18)
T3	1.36(1.08–1.72)	1.26(0.76–2.08)
*p* for trend	0.117	
Continuous scale	1.17(1.04–1.32)	1.11(0.87–1.42)
*p*-interaction	0.654	

*Adjusted for age, gender, BMI, education, income, physical activity, smoking status, drinking status, history of hypertension, diabetes, dyslipidemia, antihypertensive agents, and antidiabetic agents. Bold values: statistically significant interactions between the AC, CRI-I, and diabetes*.

## Discussion

In this large population-based study, we found that among the traditional individual lipid parameters, higher levels of TC and LDL-C were associated with the prevalence of aICAS. Several non-traditional lipid parameters, including non-HDL-C, AC, CRI-I, and CRI-II, also conveyed important information on the prevalence of aICAS and seemed to have higher predictive values than traditional individual parameters. Among all the lipid/lipoprotein biomarkers, non-HDL-C has the greatest OR value. In addition, we found that the effects of AC and CRI-I were significantly stronger in participants with diabetes than in those without.

Previous studies have indicated that dyslipidemia is a major modifiable risk factor for atherosclerosis, which is a chronic inflammatory disease that arises from imbalanced lipid metabolism and an immune response driven by the accumulation of cholesterol-laden macrophages in the artery wall (19). In the past, LDL-C was regarded as the primary biomarker of atherogenic lipoproteins and became the primary target for lipid-lowering treatments. Studies have demonstrated that LDL-C is an important causal risk factor for vascular diseases such as coronary heart disease and ischemic stroke, and its position has been reinforced, as a reduction in LDL-C with statin treatment reduced the risk of major vascular events (20). In addition to LDL-C, increased TC and TG levels were also recognized as risk factors for vascular disease, while HDL-C was considered protective (21). However, the role of traditional individual lipid parameters in ICAS is inconsistent. The Atherosclerosis Risk in Communities (ARIC) study showed that increased LDL-C and TG levels and reduced HDL-C levels were associated with the prevalence of aICAS (22). In our study, we also found that greater LDL-C was related to higher prevalence of aICAS. But inconsistent with the ARIC study, although we found that participants with aICAS seemed to have lower HDL-C levels, the difference was not statistically significant. Besides, our study demonstrated that TC was another predictor of aICAS and had a higher OR than LDL-C. The difference between the studies may be explained by the difference in ethnicity, for participants in the ARIC study were mostly White and Black, and our study targeted Chinese people. Besides, the diagnostic methods were also different, high-resolution 3T-MRA was conducted in the ARIC study, while we used TCD to detect aICAS. In another population-based study, only HDL-C, but not LDL-C, TG, or TC, had predictive value for aICAS (11). However, the recruitment in this study was limited to one rural area, and the study population was smaller, which may be the reason of the different results. Given the inconsistences in current studies, the predictive values of the traditional lipid parameters need further studies with larger population and different races.

In the optimization of the predictive capacity of the lipid profiles, in addition to traditional lipid parameters, much interest has centered on several non-traditional lipid parameters, including non-HDL-C, TG/HDL-C, AC, AIP, CRI-I, and CRI-II. Among them, non-HDL-C represents the sum of cholesterol carried by atherogenic, apolipoprotein B-containing lipoproteins, and is mostly found within LDL particles (23). It has been well-established that non-HDL-C is associated with coronary artery disease risk and the extent of coronary atherosclerosis (24, 25). In our series of APAC studies, significant associations between non-HDL-C and aICAS, asymptomatic extracranial internal carotid artery stenosis, and asymptomatic vulnerable carotid atherosclerotic plaques were found. It is noteworthy that the predictive value of non-HDL-C for aICAS is only present in men. The underlying mechanism of this result may be the potential protecting effects of estrogens on atherosclerosis development (15, 16). Furthermore, three additional well-defined atherogenic dyslipidemia parameters, AC, CRI-I, and CRI-II, were also demonstrated to be independent risk indicators for cardiovascular disease, arterial stiffness, and ischemic stroke incidence, with greater combined predictive value than either parameter used independently, which may be due to the fact that these non-traditional parameters of lipid ratios reflect the balance between proatherogenic and antiatherogenic particles (6, 9, 26, 27). A prospective study last year found that non-HDL-C and CRI-I were associated with increased coronary heart disease risk, even at low LDL-C levels, further indicating the important role of the non-traditional lipid parameters (28). In this study, we further proved that AC, CRI-I, and CRI-II were potential predictors of the prevalence of aICAS. The predictive values of the two parameters were similar, which could be explained by the fact that HDL is the difference between TC and non-HDL-C, which made AC and CRI-I have similar predict values after logarithm, indicating that these two parameters have similar values in atherosclerotic diseases.

Consistent with previous studies indicating that lipids and the related ratios are strong predictors of arterial stiffness and coronary heart disease, especially in the diabetic population (29–31), our study found interaction effects between AC and diabetes and between CRI-I and diabetes. The underlying mechanisms could be alterations in insulin-sensitive pathways, increased free fatty acid concentrations, and low-grade inflammation resulting in abnormal lipid metabolism that consistently produces a proatherogenic phenotype (32). Distorted lipid metabolism, such as decreased availability of HDL-C, participates in unloading cholesterol from the vasculature and then accelerates atherosclerosis (33). Overall, understanding the interplay between circulating lipids and the risk of diabetes and aICAS is of emerging public health importance and has implications for therapeutic development. Thus, this topic needs further exploration.

Another combined lipid parameter, the TG/HDL-C ratio, facilitates assessment of the quality and presence of abnormal lipoproteins in small, dense low-density lipoprotein, which suggests a high risk of developing diabetes (34). In addition, the TG/HDL-C ratio has a remarkable association with the estimate of insulin resistance (35). Numerous studies have demonstrated that TG/HDL-C is positively associated with the risk of atherosclerotic cardiovascular disease and ischemic stroke and could be a stronger indicator than other lipid parameters (9, 36). However, we did not observe a significant association between TG/HDL-C and aICAS in our study, as expected.

Atherogenic index of plasma, the logarithm of the molar ratio of circulating TG and HDL-C concentrations, is a simple atherogenic index and is inversely related to LDL particle size (37). Previous work supported the utility of the AIP as a stronger predictor of cardiovascular disease than other atherogenic indices, especially individual lipid parameters alone (38). But in this study, the association between the AIP and aICAS was not significant.

Overall, disentangling the associations of these lipid parameters and aICAS is of great importance to the fields of public health and clinical medicine. We observed their predictive values for the prevalence and incidence of aICAS. Finally, our results once again confirmed that two traditional lipid parameters, TC and LDL-C, were associated with the prevalence of aICAS. We further verified several non-traditional lipid parameters as novel biomarkers for aICAS, including non-HDL-C, AC, CRI-I, and CRI-II. We also found an interaction between non-traditional lipid parameters and diabetes, although the results need further study for confirmation. Our results indicate that non-traditional lipid parameters mentioned above could be used for screening people at high risk of aICAS and may be potential therapeutic targets in clinical practice, as they are all easily obtained by routine biochemical parameters.

The strengths of this study included its large population with assessment of TCD testing. However, our study has several limitations. First, it is well known that a long follow-up time is necessary to determine the causal relationship between risk factors and diseases. The APAC study is a prospective, long-term follow-up study; based on the results in this study, we will further perform a longitudinal analysis to confirm the causal relationship between these non-traditional lipid parameters and aICAS. Second, serum lipid levels may be influenced by many factors, such as diet and medication use, and participants are often encouraged to control other risk factors, such as high blood pressure, weight, and smoking. Although we excluded participants who were using lipid-lowering agents at baseline and carefully adjusted for other potential covariates in our analyses, residual confounding cannot be excluded completely. At the same time, blood lipid measurements were carried out only at baseline, but vascular damage is a complicated process that occurs over time. Third, TCD, which was the only diagnostic tool used for the detection of ICAS in this study, is partly operator dependent and prevented us from obtaining an accurate measurement of the extent of vascular stenosis data. However, TCD has been proven to be a reliable tool for detecting intracranial stenosis and shows satisfactory agreement with MRA and CTA, and it is well-suited for large-scale population screening due to its non-invasive, accessible, and affordable characteristics. Future studies could consider using other intracranial vascular diagnostic tools for the detection of ICAS to obtain more accurate information to rectify the weakness in this study. Finally, the APAC cohort targeted only Asian and middle-aged to elderly participants, which limits the generalization of our results. The generalizability of our findings to younger participants and participants of other ethnicities and races needs to be confirmed in further studies.

## Conclusions

In conclusion, this study provides novel findings that the lipid profiles of non-traditional variables, including non-HDL-C, AC, CRI-I, and CRI-II, might be considered effective markers and potential therapeutic targets for aICAS in clinical practice, especially in diabetic populations. Further prospective cohort studies with larger populations in other races and regions are needed to confirm these results.

## Data Availability Statement

The original contributions presented in the study are included in the article/Supplementary Material, further inquiries can be directed to the corresponding author/s.

## Ethics Statement

The studies involving human participants were reviewed and approved by Ethics Committee of Kailuan General Hospital (approval number: 2006-05) and Beijing Tiantan Hospital (approval number: 2010-014-01). The patients/participants provided their written informed consent to participate in this study.

## Author Contributions

JG contributed to the study concept, design, data analysis, and manuscript writing. SW and XZ had full access to all of the data in the study, take responsibility for the integrity of the data, the accuracy of the data analysis, and contributed to the data collection and analysis. AW and XZ contributed to the data collection and analysis. YW and XL contributed to the data collection. All authors read and approved the final manuscript.

## Conflict of Interest

The authors declare that the research was conducted in the absence of any commercial or financial relationships that could be construed as a potential conflict of interest.

## Publisher's Note

All claims expressed in this article are solely those of the authors and do not necessarily represent those of their affiliated organizations, or those of the publisher, the editors and the reviewers. Any product that may be evaluated in this article, or claim that may be made by its manufacturer, is not guaranteed or endorsed by the publisher.
