# Supplementary material for: Non-traditional Lipid Parameters as Potential Predictors of Asymptomatic Intracranial Arterial Stenosis
Source: Front Neurol. 2021 Aug 31;12:679415. doi: 10.3389/fneur.2021.679415 (PMC8438411; doi:10.3389/fneur.2021.679415)
Supplement: Supplementary file 1 [file Data_Sheet_1.docx]

Supplementary Material

# Supplementary Tables

| **Supplementary Table 1. Adjusted ORs with 95% confidence interval of aICAS according to non-tradition lipid parameters levels, stratified by selected risk factors** | | | | | | | | |  |
| --- | --- | --- | --- | --- | --- | --- | --- | --- | --- |
|  | Sex |  | Age |  | BMI, kg/m2 |  | Hypertension |  | |
|  | Male | Female | ≥60 | ＜60 | ＜25 | ≥25 | No | Yes | |
| TG/HDL-C |  |  |  |  |  |  |  |  | |
| T1 | 1 | 1 | 1 | 1 | 1 | 1 | 1 | 1 | |
| T2 | 1.17(0.89-1.53) | 1.49(1.06-2.10) | 1.20(0.88-1.64) | 1.32(0.99-1.76) | 1.32(1.01-1.73) | 1.09(0.78-1.53) | 1.27(0.90-1.77) | 1.20(0.92-1.58) | |
| T3 | 0.96(0.69-1.32) | 1.27(0.84-1.93) | 1.04(0.72-1.51) | 1.01(0.71-1.44) | 1.10(0.78-1.55) | 0.84(0.58-1.23) | 0.93(0.61-1.42) | 1.05(0.77-1.45) | |
| P for trend | 0.455 | 0.871 | 0.763 | 0.450 | 0.657 | 0.464 | 0.210 | 0.933 | |
| Continuous Scale | 0.99(0.87-1.31) | 1.15(0.94-1.40) | 1.03(0.86-1.24) | 1.02(0.86-1.21) | 1.08(0.92-1.28) | 0.91(0.76-1.09) | 0.99(0.81-1.21) | 1.03(0.88-1.20) | |
| P interaction | 0.767 |  | 0.895 |  | 0.637 |  | 0.630 |  | |
| AC |  |  |  |  |  |  |  |  | |
| T1 | 1 | 1 | 1 | 1 | 1 | 1 | 1 | 1 | |
| T2 | 1.03(0.77-1.38) | 1.06(0.75-1.50) | 0.83(0.58-1.19) | 1.21(0.91-1.60) | 0.90(0.68-1.21) | 1.19(0.83-1.69) | 1.04(0.72-1.48) | 1.01(0.76-1.34) | |
| T3 | 1.73(1.28-2.33) | 1.28(0.89-1.86) | 1.54(1.08-2.20) | 1.47(1.08-1.99) | 1.47(1.09-1.99) | 1.51(1.06-2.14) | 1.19(0.81-1.74) | 1.68(1.26-2.24) | |
| P for trend | ＜0.001 | 0.493 | 0.010 | 0.112 | 0.051 | 0.045 | 0.683 | 0.001 | |
| Continuous Scale | 1.34(1.16-1.56) | 1.13(0.94-1.36) | 1.28(1.07-1.54) | 1.21(1.04-1.41) | 1.20(1.03-1.41) | 1.23(1.04-1.46) | 1.09(0.90-1.32) | 1.32(1.14-1.53) | |
| P interaction | 0.060 |  | 0.170 |  | 0.376 |  | 0.659 |  | |
| AIP |  |  |  |  |  |  |  |  | |
| T1 | 1 | 1 | 1 | 1 | 1 | 1 | 1 | 1 | |
| T2 | 1.17(0.89-1.53) | 1.49(1.06-2.10) | 1.20(0.88-1.64) | 1.32(0.99-1.76) | 1.32(1.01-1.73) | 1.09(0.78-1.53) | 1.27(0.90-1.77) | 1.20(0.92-1.58) | |
| T3 | 0.96(0.69-1.32) | 1.27(0.84-1.93) | 1.04(0.72-1.51) | 1.01(0.71-1.44) | 1.10(0.78-1.55) | 0.84(0.58-1.23) | 0.93(0.61-1.42) | 1.05(0.77-1.45) | |
| P for trend | 0.638 | 0.253 | 0.636 | 0.886 | 0.470 | 0.224 | 0.378 | 0.628 | |
| Continuous Scale | 0.99(0.84-1.16) | 1.15(0.94-1.40) | 1.03(0.86-1.24) | 1.02(0.86-1.21) | 1.08(0.92-1.28) | 0.91(0.76-1.09) | 0.99(0.81-1.21) | 1.03(0.88-1.20) | |
| P interaction | 0.767 |  | 0.895 |  | 0.637 |  | 0.630 |  | |
| CRI-I |  |  |  |  |  |  |  |  | |
| T1 | 1 | 1 | 1 | 1 | 1 | 1 | 1 | 1 | |
| T2 | 1.03(0.77-1.38) | 1.06(0.75-1.50) | 0.83(0.58-1.19) | 1.21(0.91-1.60) | 0.90(0.68-1.21) | 1.19(0.83-1.69) | 1.04(0.72-1.48) | 1.01(0.76-1.34) | |
| T3 | 1.73(1.28-2.33) | 1.28(0.89-1.86) | 1.54(1.08-2.20) | 1.47(1.08-1.99) | 1.47(1.09-1.99) | 1.51(1.06-2.14) | 1.19(0.81-1.74) | 1.68(1.26-2.24) | |
| P for trend | ＜0.001 | 0.493 | 0.010 | 0.112 | 0.051 | 0.045 | 0.683 | 0.001 | |
| Continuous Scale | 1.34(1.16-1.56) | 1.13(0.94-1.36) | 1.28(1.07-1.54) | 1.21(1.04-1.41) | 1.20(1.03-1.41) | 1.23(1.04-1.46) | 1.09(0.90-1.32) | 1.32(1.14-1.53) | |
| P interaction | 0.060 |  | 0.170 |  | 0.376 |  | 0.659 |  | |
| CRI-II |  |  |  |  |  |  |  |  | |
| T1 | 1 | 1 | 1 | 1 | 1 | 1 | 1 | 1 | |
| T2 | 1.11(0.83-1.47) | 1.26(0.90-1.75) | 1.08(0.77-1.51) | 1.18(0.89-1.56) | 1.14(0.86-1.51) | 1.12(0.80-1.56) | 0.99(0.70-1.40) | 1.24(0.94-1.64) | |
| T3 | 1.53(1.16-2.01) | 1.15(0.81-1.62) | 1.72(1.25-2.37) | 1.14(0.86-1.52) | 1.34(1.00-1.79) | 1.32(0.96-1.80) | 1.06(0.74-1.52) | 1.53(1.17-2.01) | |
| P for trend | 0.003 | 0.934 | 0.007 | 0.712 | 0.034 | 0.376 | 0.986 | 0.012 | |
| Continuous Scale | 1.25(1.09-1.43) | 1.07(0.91-1.27) | 1.32(1.12-1.56) | 1.07(0.92-1.23) | 1.16(1.00-1.34) | 1.15(0.99-1.34) | 1.03(0.86-1.23) | 1.24(1.08-1.41) | |
| P interaction | 0.057 |  | 0.071 |  | 0.996 |  | 0.596 |  | |
| Adjusted for age, gender, body mass index, education, income, physical activity, smoking status, drinking status, history of hypertension, diabetes, dyslipidemia, antihypertensive agents, antidiabetic agents. BMI, body mass index; TG, triglyceride; HDL-C, high-density lipoprotein cholesterol; AC, atherogenic coefficient (calculated as non-HDL-C/HDL-C); AIP, atherogenic index of plasma (calculated as log10(TG/HDL-C)); CRI-I, castelli's risk index-I (calculated as TC/HDL-C); CRI-II, castelli's risk index-II (calculated as LDL-C/HDL-C). | | | | | | | | |  |

# Supplementary Figures


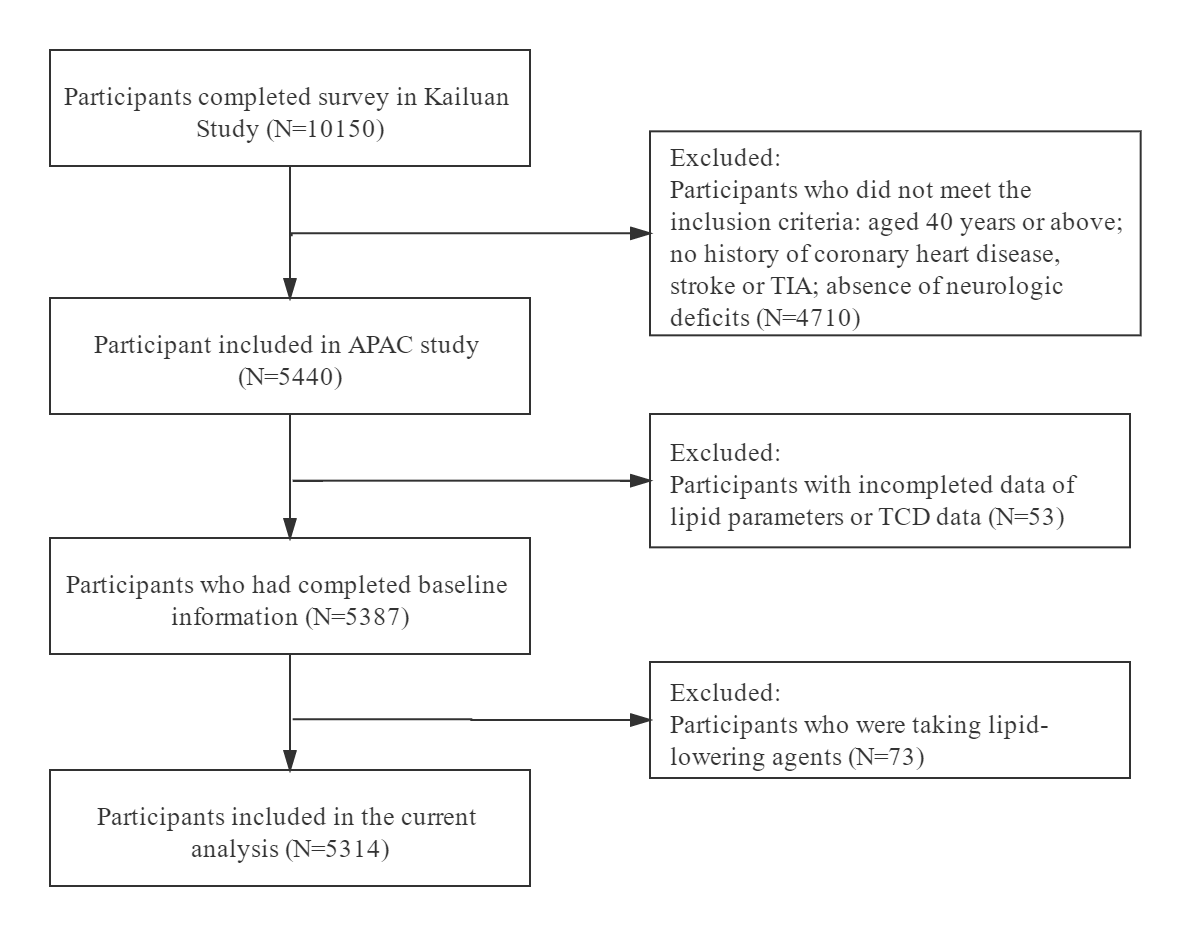


**Supplementary Figure 1.** Study flow chart.
